# DNA targeting by *Clostridium cellulolyticum* CRISPR–Cas9 Type II-C system

**DOI:** 10.1093/nar/gkz1225

**Published:** 2020-01-16

**Authors:** Iana Fedorova, Anatolii Arseniev, Polina Selkova, Georgii Pobegalov, Ignatiy Goryanin, Aleksandra Vasileva, Olga Musharova, Marina Abramova, Maksim Kazalov, Tatyana Zyubko, Tatyana Artamonova, Daria Artamonova, Sergey Shmakov, Mikhail Khodorkovskii, Konstantin Severinov

**Affiliations:** 1 Skolkovo Institute of Science and Technology, Center of life sciences, Skolkovo, Russia; 2 Peter the Great St. Petersburg Polytechnic University, Saint Petersburg, Russia; 3 National Center for Biotechnology Information, National Library of Medicine, National Institutes of Health, USA; 4 Center for Precision Genome Editing and Genetic Technologies for Biomedicine, Institute of Gene Biology, Russian Academy of Sciences, Moscow, Russia

## Abstract

Type II CRISPR–Cas9 RNA-guided nucleases are widely used for genome engineering. Type II-A SpCas9 protein from *Streptococcus pyogenes* is the most investigated and highly used enzyme of its class. Nevertheless, it has some drawbacks, including a relatively big size, imperfect specificity and restriction to DNA targets flanked by an NGG PAM sequence. Cas9 orthologs from other bacterial species may provide a rich and largely untapped source of biochemical diversity, which can help to overcome the limitations of SpCas9. Here, we characterize CcCas9, a Type II-C CRISPR nuclease from *Clostridium cellulolyticum* H10. We show that CcCas9 is an active endonuclease of comparatively small size that recognizes a novel two-nucleotide PAM sequence. The CcCas9 can potentially broaden the existing scope of biotechnological applications of Cas9 nucleases and may be particularly advantageous for genome editing of *C. cellulolyticum* H10, a bacterium considered to be a promising biofuel producer.

## INTRODUCTION

CRISPR–Cas systems are bacterial and archaeal immune systems that protect their hosts from invaders such as plasmids or bacteriophages. The immune mechanism is based on the function of Cas ribonucleoprotein effector complexes composed of Cas nucleases and CRISPR RNAs (crRNAs). crRNAs are encoded in CRISPR arrays consisting of repeats and intervening unique spacers. Some spacers are derived from invader's DNA and are introduced into CRISPR arrays during the infection. The CRISPR array is transcribed into a pre-crRNA, which is processed further to short mature crRNAs containing a single spacer and flanking repeat sequences. Complementary pairing between crRNA spacer segment and the invader genome allows Cas nucleases to specifically recognize foreign targets and degrade them, thus preventing the spread of the infection.

The crRNAs with investigator defined spacer sequences allow one to guide Cas nucleases to virtually any desirable target. Because of their relative simplicity, single-subunit Cas nucleases of Type II CRISPR–Cas systems form the basis of multiple genome editing applications. Since 2013 Type II CRISPR-based instruments are used for genome modification and transcription regulation in eukaryotic, including human, cells (1). Alongside with eukaryotic genome editing, there is a large demand for genome engineering of microorganisms useful in biotechnology and several efficient CRISPR-based methods of bacterial genome editing have been developed (2–4). Most of these genome editing approaches rely on the use of the SpCas9 protein, the most investigated to date effector nuclease from *Streptococcus pyogenes* Type II-A CRISPR–Cas system (5). Despite high DNA cleavage efficiency, SpCas9 has several limitations due to its large size, a strict requirement for an NGG PAM (protospacer adjacent motif essential for target DNA recognition) and imperfect specificity.

Bioinformatic searches for Cas9 orthologs and their subsequent biochemical characterization reveal nucleases with different properties, which can broaden Cas9 proteins application. Thus, SaCas9 from *Staphylococcus aureus* and CjCas9 from *Campylobacter jejuni*, two small size Cas9 orthologs with PAM requirements 5′- NNGRRT-3′ and 5′-NNNNRYAC-3′, respectively, were shown to be active in human cells (6,7). In 2014, Fonfara *et al.* using bioinformatics approaches detected a Type II-C system CRISPR–Cas in *Clostridium cellulolyticum* genome but no functional characterization of this system was performed (8). The mesophilic cellulolytic bacterium *C. cellulolyticum* is considered to be a promising biofuel producer since it can directly convert plant biomass to lactate, acetate, ethanol and hydrogen (9). Fast and efficient approaches of *C. cellulolyticum* genome engineering will be required to improve the fermentation properties of this microorganism. To date, several CRISPR–Cas-based strategies were applied to change the *C. cellulolyticum* genome, all of them relying on SpCas9 due to the lack of any information about the host CRISPR–Cas system (PAM requirements, guide crRNAs sequences, protospacer length etc.) (10–12). Studying of *C. cellulolyticum* Type II-C CRISPR–Cas system and, in particular, its effector Cas9 nuclease, could facilitate genome modification of this bacterium and provide an additional small-size Cas9 effector for biotechnology or biomedicine. Here, we demonstrate that *C. cellulolyticum* H10 CcCas9 protein is an active RNA-guided nuclease, which efficiently introduces double-stranded breaks in DNA targets flanked by two-nucleotide 5′-NNNNGNA-3′ PAM. To facilitate further application of CcCas9 in biotechnology, we determined the main features of this CRISPR–Cas system, such as crRNA and tracrRNA sequences, the range of temperatures required for *in vitro* activity and created a nickase version of CcCas9, which could be suitable for *C. cellulolyticum* H10 genome editing by a single-nick-assisted homologous recombination (11).

## MATERIALS AND METHODS

### Plasmids cloning

The entire predicted CRISPR–Cas Type II-C system locus of *C. cellulolyticum* including flanking regions (100 nt upstream of putative tracrRNA coding sequence and 180 nt downstream of the last DR) was PCR amplified with primers locus_F and locus_R using *C. cellulolyticum* H10 genomic DNA (DSMZ 5812) as a template. The resulting fragment was inserted into XbaI and HindIII digested pACYC184 vector using NEBuilder HiFi DNA Assembly Cloning Kit (NEB, E5520). To obtain pET21a_CcCas9 plasmid, CcCas9 coding sequence was PCR amplified with CcCas9_F and CcCas9_R primers using *C. cellulolyticum* H10 genomic DNA as a template. The resulting fragment was inserted into XhoI and NheI digested pET21a vector by NEBuilder HiFi DNA Assembly Cloning Kit (NEB, E5520). The vectors maps are presented in the Supplementary Table S1.

### Plasmid transformation interference screening

Randomized 7N plasmid libraries carried a protospacer sequence flanked by seven randomized nucleotides (Supplementary Table S1). To create the library the ssDNA oligo Library_f containing randomized nucleotides was double-stranded through single stage PCR with Library_r primer (Evrogen). This fragment was assembled with PUC19 fragment synthesized through PCR using primers PUC19_F and PUC19_R by NEBuilder HiFi DNA Assembly Cloning Kit (NEB, E5520). The mix was transformed to *Escherichia coli* DH5alpha strain and plated to media supplemented with 100 μg/ml ampicillin. The plates were incubated at 37°C. Eighteen hours after transformation >50 000 colonies were washed off the plates, and the plasmid library was extracted by Qiagen Plasmid Maxi kit (Qiagen 12162). HTS analysis of the library showed representation of 15716 PAM variants. The library plasmid map is presented in the Supplementary Table S1. Competent *E. coli* Star cells carrying pACYC184_CcCas9_locus or an empty pACYC184 vector were transformed with 7N PAM plasmid libraries and plated to 100 μg/ml ampicillin and 25 μg/ml chloramphenicol containing agar plates. After 16 h, cells were harvested and DNA was extracted using Qiagen Plasmid Maxi kit (Qiagen 12162). PAM-containing sequences were PCR amplified using M13_f and M13_r primers and sequenced using Illumina platform with pair-end 150 cycles (75 + 75).

### Bacterial RNA sequencing

*E. coli* DH5alpha carrying pACYC184_CcCas9_locus were grown 16 h at 37°C in LB (Luria Bertani) medium supplemented with 25 μg/ml chloramphenicol. Bacteria were resuspended in TRIzol (ThermoFisher, 15596026). Total RNA was purified using Direct-Zol RNA kit (Zymo research, R2051). RNA was DNase I (Zymo research) treated and 3′ dephosphorylated with T4 PNK (NEB, M0201). Ribo-Zero rRNA Removal Kit (Gram-Negative Bacteria) kit (Illumina, 15066012) was used to remove ribosomal RNA. HTS samples were prepared using NEBNext Multiplex Small RNA Library Prep Set for Illumina (NEB, E7300). The library was sequenced using Illumina platform with pair-end 150 cycles (75 + 75).

### RNA sequencing analysis

HTS results of RNA sequencing were aligned to the reference plasmid pACYC184_CcCas9_locus using BWA aligner (13). Determined coordinates of 5′ and 3′ RNA ends were used to reconstruct the full-length RNA sequences. The resulting fragments were analyzed using Geneious 11.1.2. Filtered 40–130 nt-length sequences were used to generate the alignment.

### *In vitro* DNA cleavage assays

DNA cleavage reactions were performed using the recombinant CcCas9 protein and linear dsDNA targets. The reaction conditions were: 1× CutSmart (NEB, B7204) buffer, 1 mM DTT, 30 nM DNA, 400 nM CcCas9, 2 μM crRNA, 2 μM tracrRNA. Samples were incubated at an appropriate temperature for 20 min (unless otherwise stated). Further, 4× loading dye containing 10 mM Tris–HCl, pH 7.8, 40% glycerol, 40 mM EDTA, 0.01% bromphenol blue, 0.01% xylene cyanol was added to stop the reaction. Reaction products were analyzed by electrophoresis in 1.5% agarose gels or, where indicated, in 1× TBE polyacrylamide gels. Pre-staining with ethidium bromide or post-staining with SYBR gold stain (ThermoFisher, 11494) was used for visualization of bands on agarose or polyacrylamide gels, correspondingly.

All *in vitro* DNA cleavage reactions were performed at 45°C unless otherwise stated. For testing the activity of CcCas9 at different temperatures a mix of CcCas9 protein with *in vitro* transcribed crRNA–tracrRNA in the cleavage buffer, and the DNA substrates, also in the cleavage buffer, were first incubated separately at the chosen temperature for 10 min, combined, and incubated for additional 10 min at same temperature.

For *in vitro* PAM screens, 100 nM linear DNA 7N PAM library was incubated with 400 nM CcCas9, 1 μM crRNA and 1 μM tracrRNA. Reactions without crRNA were used as negative controls. The reactions were performed at 45°C for 20 min. Reaction products were separated by electrophoresis in agarose gels. Uncleaved DNA fragments were extracted from the gel using Zymo Clean Gel Recovery kit (Zymo research, D4007). HTS libraries were prepared using Ultra II DNA library prep kit (NEB, E7646). Samples were sequenced using MiniSeq Illumina with single-end 150 cycles. All RNAs used in this study are listed in Supplementary Table S2.

### Computational sequence analysis

For PAM screens results analysis, Illumina reads were filtered by requiring an average Phred quality (*Q* score) of at least 20. Resulting reads were mapped against the corresponding reference sequence using BWA (13). All unmapped reads were discarded from the analysis. The degenerate 7-nucleotide region was extracted from the sequences.

For interference PAM screens analysis, depleted PAM sequences were determined by comparing the number of each PAM counts for CRISPR CcCas9 sample and control. The representation of unique PAM in both samples, as well as PAM representation of initial 7N library was >15 000 PAM variants. WebLogo was used to generate a logo based on 887 of statistically significantly (one-sided Pearson chi-square test with a *P*-value < 10^−12^) depleted PAM sequences (listed in Supplementary File S2). In case of *in vitro* PAM determination screens 16 364 and 16 363 unique PAM sequences were found, respectively, for the depleted and control samples. Depletion values of PAM sequence positions were counted according to (14). The frequencies of each PAM variants in depleted and control samples were processed by R script. The frequencies of PAM variants were also used for PAM wheel construction.

### Recombinant protein purification

For recombinant CcCas9 purification competent *E. coli* Rosetta cells were transformed with pET21a_CcCas9 plasmid and grown till OD_600_ = 0.6 in 500 ml LB media supplemented with 100 μg/ml ampicillin. The target protein synthesis was induced by the addition of 1 mM IPTG. After 18 h of growth at 22°C, cells were centrifuged at 4000g, the pellet was resuspended in lysis buffer containing 50 mM Tris–HCl pH 8.0 (4°C), 500 mM NaCl, 1 mM β-mercaptoethanol and 10 mM imidazole supplemented with 1 mg/ml lysozyme (Sigma) and cells were lysed by sonication. The cell lysate was centrifuged at 16 000g (4°C) and filtered through 0.45 μm filters. The lysate was applied to 1 ml HisTrap HP column (GE Healthcare) and CcCas9 was eluted by imidazole gradient in the same buffer without lysozyme. After affinity chromatography, fractions containing CcCas9 were applied on a Superose 6 Increase 10/300 GL (GE Healthcare) column equilibrated with a buffer containing 50 mM Tris–HCl pH 8.0 (4°C), 500 mM NaCl, 1 mM DTT. Fractions containing CcCas9 monomer were pooled and concentrated using 30 kDa Amicon Ultra-4 centrifugal unit (Merc Millipore, UFC803008). Glycerol was added to final concentration of 10% and samples were flash-frozen in liquid nitrogen and stored at −80°C. Purity of CcCas9 was assessed by denaturing 8% PAGE and the integrity of recombinant protein was confirmed by mass spectrometry.

## RESULTS AND DISCUSSION

### *Clostridium cellulolyticum* H10 CRISPR–Cas II-C system: locus organization

**Figure 1.**
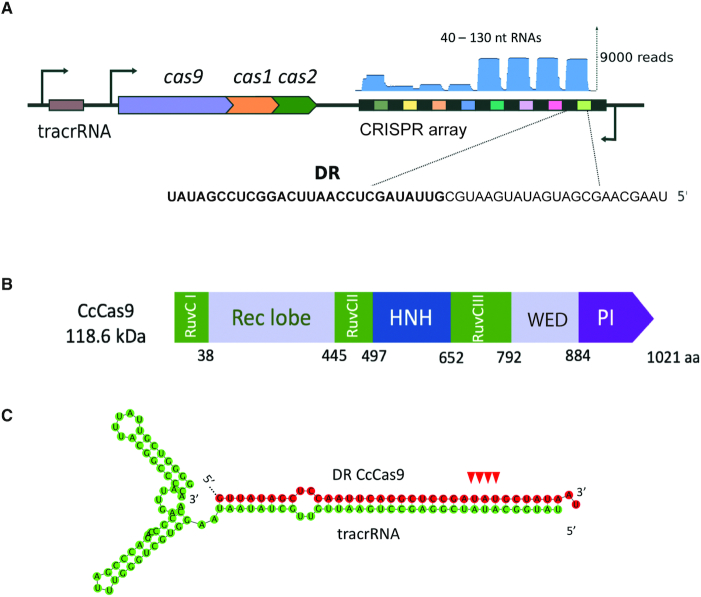
Organization of *Clostridium cellulolyticum* H10 CRISPR–Cas Type II-C locus. (**A**) A scheme of the *C. cellulolyticum* H10 CRISPR–Cas locus. DRs (direct repeats) are shown as black rectangles, spacers are indicated by rectangles of different colors. The tracrRNA coding sequence is shown as a brown rectangle. The *cas* genes are labeled. The direction of transcription is indicated with black arrows. Mapping of small RNAs reads revealed by RNA-seq is shown at the top of CRISPR array in blue. A sequence of typical mature crRNA is expanded below with the DR part shown in bold typeface. (**B**) Domain organization of the CcCas9 protein. (**C**) *In silico* co-folding of *C. cellulolyticum* H10 CRISPR–Cas Type II-C system DR and putative tracrRNA. The DR sequence is colored in red, the tracrRNA sequence is colored in green. The cleavage sites introduced during crRNA maturation are indicated with red arrows. Co-folding was performed using Geneious software, free energy of structure shown is −80.50 kcal/mol.

The *C. cellulolyticum* H10 type II-C CRISPR–Cas locus was bioinformatically found by Fonfara *et al.* in 2014 but up to date there is no information about the activity of this system. The CRISPRFinder tool (https://crispr.i2bc.paris-saclay.fr/Server/) revealed an array composed of nine 36-bp DRs (direct repeats) interspaced by 31-bp spacers in the proximity of the *cas* genes operon (Figure 1A). A Blast search using spacer sequences as queries revealed no matches to sequences from publicly accessible databases. The *C. cellulolyticum* H10 *cas* genes comprise the CcCas9 effector nuclease gene and the adaptation module composed of *cas1* and *cas2* genes. Being a II-C type Cas nuclease, CcCas9 has a relatively small size (1021 amino acids or 118 kDa) compared to the widely used SpCas9 (1368 amino acids/158 kDa). Alignment of the CcCas9 amino acid sequence with the previously characterized small-size Type II-A SaCas9 protein from *S. aureus* shows the presence of all domains necessary for nuclease activity (Figure 1B, Supplementary Figure S1). Upstream of *cas* genes, we identified a putative tracrRNA-encoding sequence with an anti-repeat partially complementary to DRs. *In silico* co-folding of part of DR with the putative tracrRNA predicts a stable secondary structure (Figure 1C).

The entire CRISPR–Cas locus of *C. cellulolyticum* H10 with adjacent non-coding sequences likely containing promoters was cloned into *E. coli* pACYC184 plasmid vector for heterologous expression. Although *E. coli* cells carry a CRISPR–Cas system of their own, it belongs to a different class (type I-E), relies on different kinds of crRNAs, and is inactive at least at laboratory conditions (15). Thus, no influence of resident CRISPR–Cas on the function of *C. cellulolyticum* H10 CRISPR–Cas is expected. To determine the polarity of *C. cellulolyticum* H10 CRISPR array transcription and confirm the tracrRNA sequence, small RNAs present in *E. coli* heterologously expressing *C. cellulolyticum* H10 CRISPR–Cas locus were sequenced. We found that the CRISPR array is actively transcribed in the orientation opposite to the *cas* genes transcription and mature crRNAs corresponding to every spacer in the array could be detected (Figure 1A). This could be due to efficient processing of pre-crRNA or, alternatively, due to transcription from internal promoters embedded into the repeat sequence, as has been observed in some Type II-C systems (16). Indeed, we noted that the terminal nine nucleotides of *C. cellulolyticum* H10 DRs have a sequence similar to bacterial extended −10 promoter consensus element, as is also the case for *Neisseria meningitidis* CRISPR–Cas II-C system, where transcription initiation within each repeat has been shown experimentally (16). Each *C. cellulolyticum* H10 crRNA contains 23–26 nt of spacer sequence and 24–28 nt of DR. The tracrRNA coding sequence is also expressed, generating variably sized, 70–107 nt, products. In the natural host, the length of mature crRNAs and tracrRNA could be slightly different from those obtained during heterologous expression in *E. coli*.

### Determination of CcCas9 PAM by DNA interference screening

**Figure 2.**
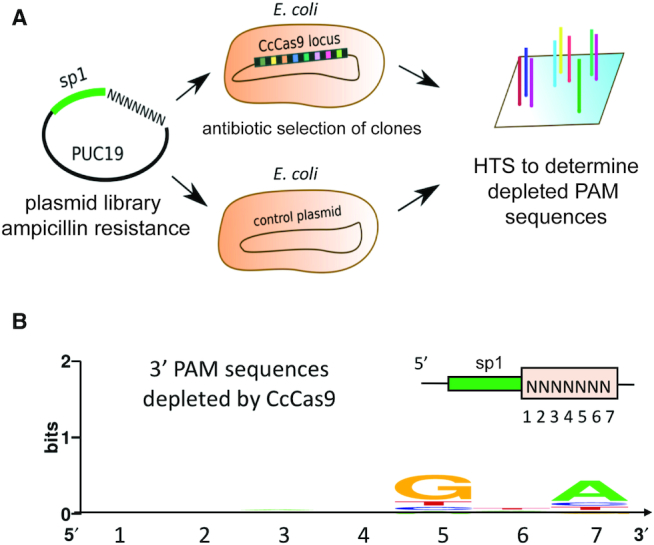
Determination of CcCas9 PAM sequence using plasmid transformation interference screening. (**A**) A scheme of the bacterial interference screen experiment. *Escherichia coli* cells carrying the CcCas9 locus were transformed by PUC19-based library carrying the protospacer sequence flanked by seven randomized nucleotides and plated on ampicillin containing plates. The presence of an interference-proficient PAM decreases the frequency of plasmids with this PAM among ampicillin-resistant colonies. Comparison of PAM representation in CcCas9 locus carrying cells and in control cells without the CcCas9 locus reveals depleted PAM sequences and allows one to deduce the PAM consensus. (**B**) *Clostridium cellulolyticum* CRISPR–Cas Type II-C system PAM sequence logo determined by plasmid transformation interference screening.

Given robust expression of *C. cellulolyticum* crRNAs in *E. coli*, we performed a bacterial interference screen to determine the CcCas9 protospacer adjacent motif (PAM) sequence (Figure 2A). Based on the knowledge about organization of known Cas9-guide RNAs–target DNA complexes and the direction of *C. cellulolyticum* CRISPR array transcription, we designed a plasmid-based PAM library carrying a 30-bp protospacer sequence matching the first spacer in the *C. cellulolyticum* CRISPR array flanked at one side with seven randomized nucleotides (Figure 2A). *E. coli* cells carrying a compatible plasmid with the CcCas9 locus or an empty vector were transformed with the library and plated on a medium that only allowed the growth of cells carrying both plasmids. High-throughput sequencing of the targeted protospacer region amplified from plasmids extracted from pooled transformant colonies revealed depletion of 887 out of 16 384 library members in cells carrying the CcCas9 locus compared to control cells (Supplementary File S2). Most of depleted variants had a 5′-NNNNGNA-3′ sequence, indicating that CcCas9 prefers purines at positions 5 and 7 of the non-target DNA strand downstream of the protospacer (Figure 2B).

### *In vitro* cleavage of DNA by CcCas9

**Figure 3.**
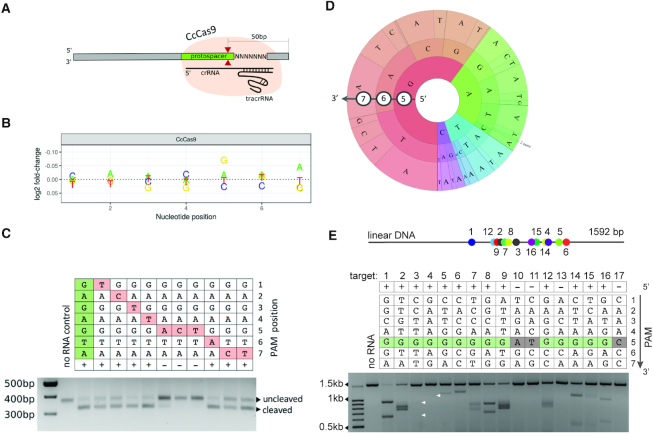
*In vitro* cleavage of DNA targets by CcCas9. (**A**) Scheme of the DNA library used for *in vitro* PAM screening experiment. Cleavage of 7N DNA library with CcCas9 generates DNA products shortened by 50 bp. The location of the cleavage site is shown with red arrows. (**B**) Analysis of depletion of PAM library sequences after *in vitro* cleavage. (**C**) Single-nucleotide substitutions in the 5th position of PAM prevent DNA cleavage by CcCas9. An agarose gel showing the results of electrophoretic separation of cleavage products of targets with PAM sequences shown at the top is presented. The +/– signs signify, correspondingly, whether cleavage was or was not observed. Bands corresponding to cleaved and uncleaved DNA fragments are indicated. (**D**) Wheel representation of *in vitro* PAM screen results for fifth, sixth and seventh nucleotide positions of PAM. Nucleotide positions from the inner to outer circle match the PAM positions moving away from the protospacer. For a given sequence, the area of the sector in the PAM wheel is proportional to the relative depletion in the library. (**E**) *In vitro* cleavage of different 20-bp target sites on a linear DNA fragment by CcCas9. The PAM sequences corresponding to each target are shown in the table. The +/– signs signify, correspondingly, whether cleavage was or was not observed. The conserved G at the fifth position is indicated by green color. Below, a gel showing results of *in vitro* cleavage of targets with indicated PAMs is presented. White arrows indicate positions of poorly visible bands. Above, a scheme showing the relative positions of the targeted DNA sites on the linear DNA fragment is presented.

Based on the interference screening experiments results we proceeded to reconstitute CcCas9 DNA cleavage *in vitro*. A recombinant CcCas9 was purified (Supplementary Figure S2) and tested for its ability to cleave linear DNA PAM libraries containing a target site flanked with seven randomized nucleotides (Figure 3A). Since *C. cellulolyticum* H10 was isolated from decayed grass in a compost pile (17), we first performed DNA cleavage reactions at 33°C, the reported optimal growth temperature (17), but did not detect any cleavage. The change of reaction temperature to 45°C led to observable library DNA cleavage. Uncleaved DNA fragments as well as a negative control (original DNA PAM library incubated with DNA cleavage reaction components in the absence of crRNA) were sequenced using the Illumina platform. Comparison of PAM variants representation in experimental and control samples allowed us to determine PAM sequences depleted in the presence of the CcCas9 effector complex. The analysis revealed that recombinant CcCas9 in complex with *in vitro* synthesized tracrRNA and crRNA was able to cleave DNA targets with ‘NNNNGNA’ PAM at the 3′-flank, in agreement with results obtained during *in vivo* interference screening (Figure 3B), although A at the 7th position was less conserved comparing to G at the 5th position.

To validate CcCas9 PAM sequence preferences, single-nucleotide substitutions in the deduced consensus PAM sequence were introduced and individually tested for cleavage efficiency (Figure 3C). The results confirmed the importance of a G at the fifth position and a less strict preference for an A at the seventh position (Figure 3C). To further investigate CcCas9 PAM sequence preferences, in particular, to identify individual sequences representing functional PAMs and the relative activity of each sequence, we used the PAM wheel approach developed by Leenay *et al.* (18) for results visualization. The PAM wheel confirmed the 5′-NNNNGNA-3′ motif with a moderate preference for an A at the seventh position but also revealed a slight bias for an A in addition to G in the fifth position (Figure 3D).

We next tested CcCas9 DNA cleavage activity on different targets flanked by the 5′-NNNNGNA-3′ consensus PAM as well as 5′-NNNNGNN-3′ PAM sequences (Figure 3E). Several 20-bp target sites with CcCas9 PAM in a 1592-bp PCR fragment of human grin2b gene were selected, the corresponding crRNAs synthesized, and *in vitro* cleavage reactions were performed with recombinant CcCas9 charged with these crRNAs. Control crRNAs recognizing sequences flanked by PAMs with no G at the fifth position were also tested. As can be seen from Figure 3E, CcCas9 did not recognize targets flanked by control sequences with substitutions of G at the fifth position. On the other hand, the CcCas9 nuclease recognized and cleaved not only targets with 5′-NNNNGNA-3′ consensus PAM, but also targets flanked by 5′-NNNNGNN-3′ sequences, confirming that 5′-NNNNGNN-3′ PAMs are functional. Similar results were obtained when in *in vitro* DNA cleavage by CcCas9 was performed using a supercoiled plasmid carrying the cloned grin2b gene fragment (Supplementary Figure S3). The cleavage efficiency of CcCas9 on different DNA targets varied significantly, which is likely a combination of contributions by protospacer sequences and by identity of ‘N’ nucleotides in the PAM. Overall, based on plasmid transformation interference screening results and *in vitro* DNA cleavage data, we conclude that CcCas9 recognizes a two-nucleotide 5′-NNNNGNA-3′ PAM, with requirement for an A in seventh position being not very stringent. To the best of our knowledge, this PAM is distinct from PAM sequences of known Cas9 nucleases.

**Figure 4.**
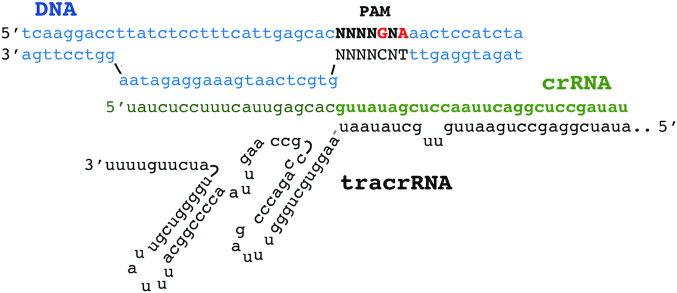
A scheme of the CcCas9 DNA–cleavage complex. DNA is shown in blue, crRNA in green and tracrRNA in black.

Experiments described above were conducted using the tripartite system composed of CcCas9, crRNA and tracrRNA. To simplify the CcCas9 DNA-cleavage process, we sought to design sgRNA, a single guide RNA where crRNA is fused to tracrRNA. Several sgRNA variants were tested, but none were active *in vitro* (Supplementary Figure S4). Thus, the CcCas9 DNA minimal cleavage system to date consists of three components: CcCas9 nuclease, tracrRNA and crRNA (Figure 4). Additional studies might reveal the requirements for a functional sgRNA sequence in this system.

One of the possible applications of CcCas9 is genome modification of its host, *C. cellulolyticum*. To facilitate further use of CcCas9 for editing of *C. cellulolyticum* via the single-nick-assisted HR strategy proposed by Xu *et al.* (11), we generated a CcCas9 nickase version by mutating the aspartic acid D8 to alanine in the active site of CcCas9 RuvC nuclease domain. The incubation of D8A CcCas9 mutant with a double-stranded DNA target in the presence of crRNA and tracrRNA led to cleavage of only one DNA strand, as expected (Supplementary Figure S5).

### Activity of CcCas9 at different temperatures

**Figure 5.**
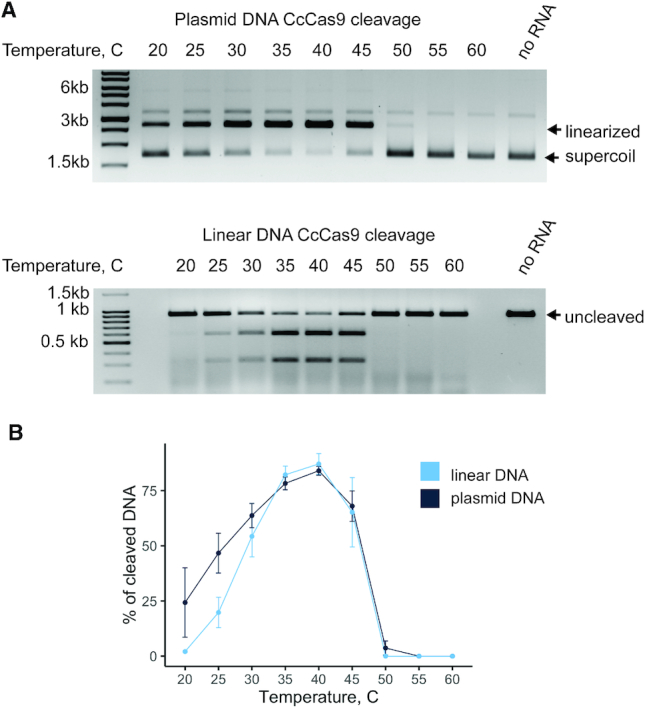
Activity of CcCas9 at different temperatures. (**A**) CcCas9 was incubated with tracrRNA, crRNA and a 2.7 kb plasmid DNA (above) or a 921 bp linear DNA fragment (below) containing a target sequence at indicated temperatures for 10 min. Products were separated by agarose gel electrophoresis. (**B**) CcCas9 was incubated with tracrRNA, crRNA and plasmid or linear DNA as in panel A. Cleavage efficiency (in per cent) was calculated as a ratio of intensity of staining of cleaved bands to the combined intensity of cleaved and uncleaved bands. Mean values and standard deviations obtained from three independent experiments are shown.

Based on the initial observations showing that DNA cleavage by CcCas9 is temperature-dependent, we decided to determine the dependence of its nuclease activity on temperature. Incubation of CcCas9, crRNA, tracrRNA and plasmid carrying a protospacer flanked by consensus PAM sequence 5′-ACAGGTA-3′ at different temperatures led to efficient cleavage of the target in a temperature range of 25–45°C with maximal cleavage at 40°C (Figure 5A and B). CcCas9 cleavage of a linear DNA fragment carrying the same target site showed similar temperature activity profile.

**Figure 6.**
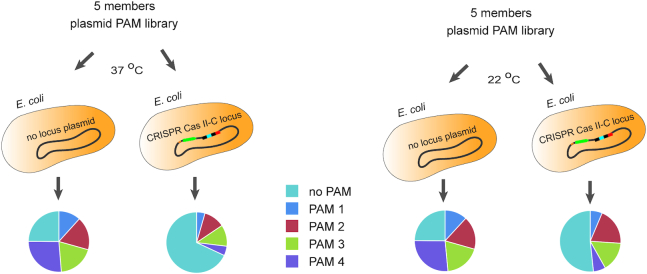
The influence of temperature on *Clostridium cellulolyticum H10* CRISPR–Cas II-C locus interference. A plasmid library composed of five members carrying protospacer matching the first spacer in the CRISPR array and flanked by 5′-ACAGGTA-3′(PAM 1), 5′-CGGTGTA-3′ (PAM 2), 5′-TGAAGAA-3′ (PAM 3), 5′-ATTGGAA-3′ (PAM 4), and 5′-TTCATAT-3′ (no PAM) sequence was transformed in *E. coli* cells carrying a plasmid with the *C. cellulolyticum H10* CRISPR–Cas II-C locus or a control plasmid. Cells were plated on LB media supplemented with ampicillin and chloramphenicol and grown for 18 h at 37°C (left panel) or 22°C (right panel). The plasmid DNA was extracted from grown colonies and HTS was used to estimate the representation of each library member. The pie charts showing PAM representation in colonies formed are shown below. Each colored sector represents a fraction of corresponding PAM sequence.

Given the observed differences in CcCas9 *in vitro* DNA cleavage efficiency at room temperature and at 37°C, we compared the CcCas9 CRISPR–Cas II-C system interference activity at 22°C and 37°C. To this end, we used an equimolar mixture of five PUC19-based plasmids carrying a protospacer matching the first spacer in the CRISPR array and flanked by 5′-ACAGGTA-3′, 5′-CGGTGTA-3′, 5′-TGAAGAA-3′ and 5′-ATTGGAA-3′ CcCas9 PAM variants and a 5′-TTCATAT-3′ sequence as a ‘no PAM’ control. This 5-members PAM library was transformed into competent *E. coli* cells carrying pACYC184_CcCas9_locus plasmid or pACYC184 vector as a control. Cells were plated on LB medium supplemented with ampicillin and chloramphenicol and grown for 18 h at either 22 or 37°C. Plasmid DNA was purified from colonies formed at each temperature and HTS of PAM-containing regions was performed to determine the changes in representation of library members (Figure 6, Supplementary Table S3). Analysis of HTS results showed the decrease in the frequency of 5′-NNNNGNA-3′ PAM-containing plasmids in cells carrying the CcCas9 locus due to interference and corresponding increase of the ‘no PAM’ plasmid representation at 37°C as well as at 22°C compared to control (Supplementary File S1, Supplementary Figure S6). The observed effect was stronger in colonies formed at 37°C than at 22°C. Plasmids with different 5′-NNNNGNA-3′ PAM sequences showed different depletion levels. Thus, the temperature dependence of *C. cellulolyticum* CRISPR–Cas II-C system can be observed in bacteria as well as *in vitro*.

## CONCLUSION

Despite the extensive use of Cas9 nucleases for genome engineering, to date, only several Cas9 orthologs can be considered as well-characterized. Given the diversity of Type II CRISPR–Cas systems, Cas9 orthologs can show significant variations in PAM requirements, specificity and other biochemical properties. In this work, we functionally characterized CRISPR–Cas system from *Clostridium cellulolyticum* H10. When introduced in *E. coli*, the *C. cellulolyticum* CRISPR–Cas system shows high levels of crRNA expression, as well as interference against plasmid transformation. The *C. cellulolyticum* Cas9 effector, CcCas9, is a Type II-C endonuclease and thus has a relatively small (compared to other Type II effector proteins) molecular weight. This nuclease in complex with tracrRNA and crRNA actively cleaves DNA targets flanked by two-nucleotide PAM sequence 5′-NNNNGNA-3′. Most other small Type II-C Cas9 effectors have more complex PAM requirements, i.e. NmeCas9, CjeCas9 and GeoCas9 require, 5′-NNNNGNTT-3′, 5′-NNNNRYAC-3′ and 5′-NNNNCNAA-3′, respectively (7,19–20). The simple, two-nucleotide PAM of CcCas9 may thus be considered as an advantage for future biotechnology applications. Whereas further studies are needed to check the ability of CcCas9 to edit eukaryotic genomes, we envision that the CRISPR–Cas system characterized here potentially can be conveniently used as an instrument for *C. cellulolyticum* H10 genome engineering.

## DATA AVAILABILITY

Raw sequencing data have been deposited with the National Center for Biotechnology Information Sequence Read Archive under BioProject ID PRJNA554628.

## Supplementary Material

gkz1225_Supplemental_Files
