# Supplementary material for: DNA targeting by Clostridium cellulolyticum CRISPR–Cas9 Type II-C system
Source: Nucleic Acids Res. 2020 Jan 16;48(4):2026–34. doi: 10.1093/nar/gkz1225 (PMC7038990; doi:10.1093/nar/gkz1225)
Supplement: gkz1225_Supplemental_Files [file gkz1225_supplemental_files.zip › Supplementary File S1_R2.pdf]

Supplementary File S1

Figure S1. CcCas9 and SaCas9 protein alignment. The putative protein domains are shown by colored blocks.

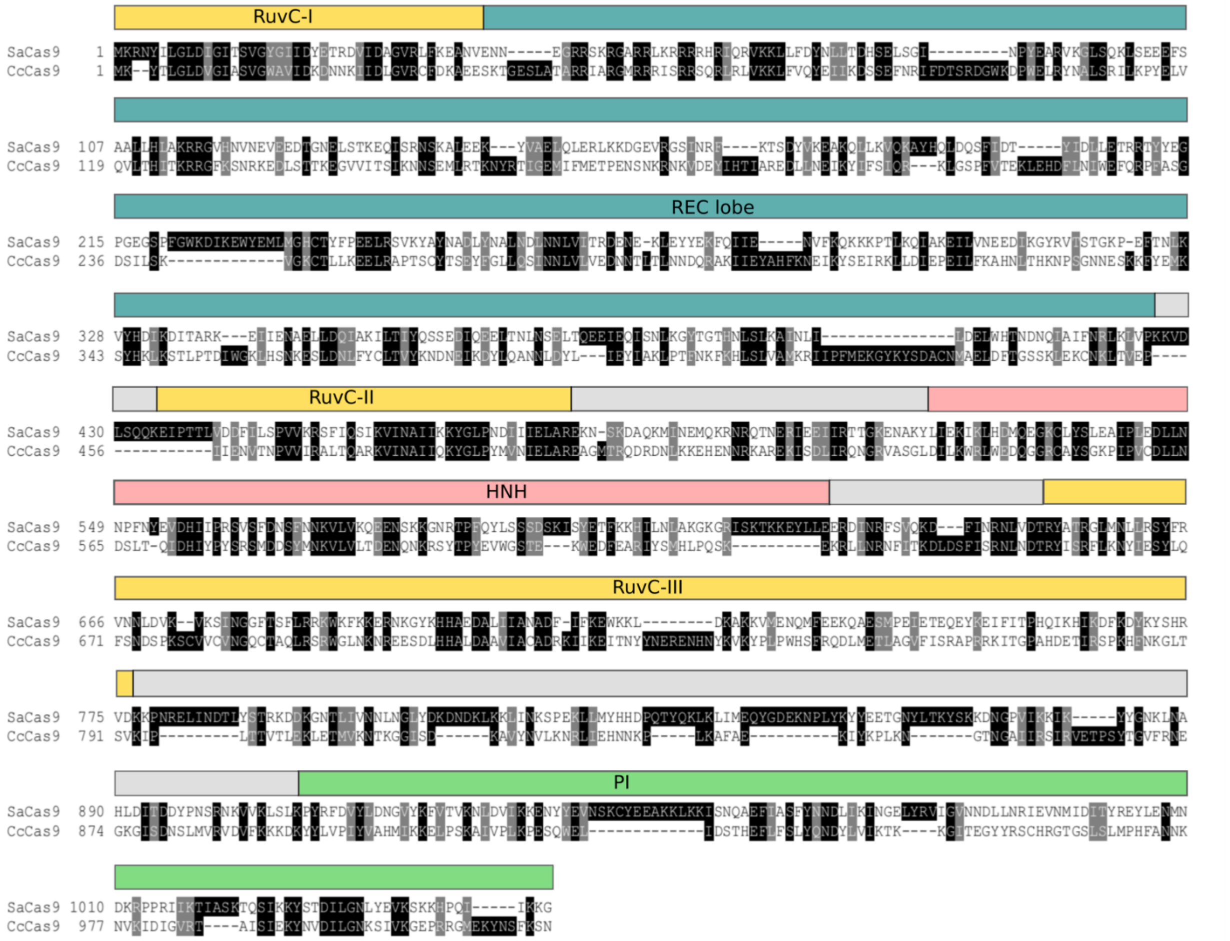

Table S1. DNA sequences used in this study.

PUC19 7N fragment	ctatgaccatgattacgccaagctNNNNNNNGCATTCATATCATCGCTTGCTTATTTTTTA<br>cccgggtaccgagctcga
	https://benchling.com/s/seq-9AUgIqLVZMC8P2fi0fHy
Pacyc184_CcCas9_locus plasmid	https://benchling.com/s/seq-JtQiuWbwPZSgoYDPVQdo
pET21a_CcCas9	https://benchling.com/s/seq-vDHDkNAp45fykKGHOVCk
Plasmids with different CcCas9 target sites (PUC19 with human grin2b gene fragment)	https://benchling.com/s/seq-UBYcoVMlXuLO5PUYrmMc
DNA fragment with different CcCas9 target sites (human grin2b gene fragment)	https://benchling.com/s/seq-FvBzwSlYhkYoQthrlbZw
	GAGAGAGATGGCCAAGGCTTATATTCTATAGAGCATTATGTCCTTAGTTTGATGCATAGAA<br>TAAGATTTAGGGTCATATGTGGAAGTAAAAAGGAAGGAGTTCTTTGTAGGTAAAAGGTGGC<br>AAATTATATGAAAATACGGTATCAGTCATTTTAGGGAAGTCACGACTATAGGATGGCATCA<br>GGAAAAAAAAAGGAACATTTTTCAAATGTGGCTCTAACATTACTTCAGCTGCTAATGGTAT<br>TTGTTTAAGTTTCTGTATTTTGGTGTATAAATAGATTGGAGTAATATGTGTTCCTTATAAT<br>AATTGGTTATATGAGAGGCAGTTCCACGTAGTGTAATAGAACACATATTGGAATACAAAAG<br>TCAGAAGATCTGGGTTCAGGTTTGTTTCACTTAATTGATTGGTTCATGGTCTTAGAACACT<br>TAGCTTCTCTGAGCCTTGGCGTCAACATTTATAAAAATGGTGATAATAATGTTTTTCTTAT<br>TTTATTCCCTACTGGGTCATTGTAAGGATCAATTGAGGCAATGTTTTAAAACTACTAGTCA<br>TGTATCAGTTGTTCTTGTAGTTTAATATTAAGAGCCAGATACTAACAAGGTTACTAAAGAA<br>TTTTCTGGCTGTTGTCCTCATTGAGGCAAACATAAGGTGAAGGCAGCAAGAATGCAGGGCT<br>TGTGTACTTATAGCCCCCCACATCCAGTTTATCCAGCCCATGTTCTGTTGCTCACCTCTGC<br>TGAGCACGTTTTTCTGCTCACTTTGTCTGGCCTTGCTTTCCTTCAGCCCAAGAACAGTACA<br>AGGGTGGGCTGTAACAGGAGGGCCAGGAGATTTGTGTATGCATACTCGCATGGCTACCTGG<br>ACCACTCACAACCTCTTTTCCTCCTTTGTCTCTGCCTGTAGCTGCCAATGACTATAGCAAT<br>AGCACCTTTTATTGCCTTGTTCAAGGATTTCTGAGGCTTTTGAAAGTTTCATTTTCTCTCA<br>TTCTGCAGAGCAAATACCAGAGATAAGAGAGTAGGCTGGTAGATGGAGTTGGGTTTGGTGC<br>TCAATGAAAGGAGATAAGGTCCTTGAATTGCAGTATCTAGCCTCTTCTAAGACAGGTTACG<br>TGATGTAGATCCTATTTTAACATGCTCTTTCTTTGTGTTTGCAGGGAGTCGACGAGTTGAA<br>GATGAAGCCCAGAGCGGAGTGCTGTTCTCCCAAGTTCTGGTTGGTGTTGGCCGTCCTGGCC<br>GTGTCAGGCAGCAGAGCTCGTTCTCAGAAGAGCCCCCCCAGCATTGGCATTGCTGTCATCC<br>TCGTGGGCACTTCCGACGAGGTGGCCATCAAGGATGCCCACGAGAAAGATGATTTCCACCA<br>TCTCTCCGTGGTACCCCGGGTGGAACTGGTAGCCATGAATGAGACCGACCCAAAGAGCATC<br>ATCACCCGCATCTGTGATCTCATGTCTGACCGGAAGATCCAGGGGGTGGTGTTTGCTGATG<br>ACACAGACCAGGAAGCCATCGCCCAGATCCTCGATTTCATTTCAGCACAGACTCTCACCCC<br>CATCCTGGGCATCCACGGGGGCTCCTCTATGATAATGGCAGATAAGGTAAAAAGGGGCTGC<br>AGGGAG

	target site	PAM	DNA cleavage products
target1	TGAGGCAAACATAAGGTGAA	GGCAGCA	648bp + 944bp
target2	TAACAGGAGGGCCAGGAGAT	TTGTGTA	821bp + 771bp
target3	AGCAATAGCACCTTTTATTG	CCTTGTT	926bp + 666bp
target4	CGACTCCCTGCAAACACAAA	GAAAGAG	1132bp + 460bp
target5	ACGGCCAACACCAACCAGAA	CTTGGGA	1196bp + 396bp
target6	GAACGAGCTCTGCTGCCTGA	CACGGCC	1226bp + 366bp
target7	GGAAAAGAGGTTGTGAGTGG	TCCAGGT	858bp + 734bp
target8	TATAGTCATTGGCAGCTACA	GGCAGAG	893bp + 699bp
target9	TGTAACAGGAGGGCCAGGAG	ATTTGTG	819bp + 773bp
target10	CTACATCACGTAACCTGTCT	TAGAAGA	-
target11	TCCGCTCTGGGCTTCATCTT	CAACTCG	-
target12	ACAAGGGTGGGCTGTAACAG	GAGGGCC	807bp + 785bp
target13	CACCAACCAGAACTTGGGAG	AACAGCA	-
target14	ATCTACATCACGTAACCTGT	CTTAGAA	1091bp + 501bp
target15	AAGAGGCTAGATACTGCAAT	TCAAGGA	1066bp + 526bp
target16	GATAAGAGAGTAGGCTGGTA	GATGGAG	1014bp + 578bp

	target17 TATCTCCTTTCATTGAGCAC CAAACCC -

Locus_F	caagaagatcatcttattaatcagataaaatatttctagaTATGGTAGCAAATATGAATGTAAAGTG
Locus_R	tagcaatttaactgtgataaactaccgcattaaagcttGTACTATTTGAGGGTCGTAGTTTGTGGATATAATTTC
CcCas9_F	aaggagatatacatatggctagcATGAAATATACATTAGGTCTTGATGTTG
CcCas9_R	tggtggtggtggtgctcgagGTTGGATTTGAAACTATTATATTTCTCCATCCCACG
PUC19_F	cccgggtaccgagctcga
PUC19_R	agcttggcgtaatcatggtcatag
Library_f	ctatgaccatgattacgccaagctNNNNNNNGCATTCATATCATCGCTTGCTTATTTTTTAcccgggtaccgagctcga
Library_r	tcgagctcggtacccgggt
M13_f	GTTGTAAAACGACGGCCAGTG
M13_r	AGCGGATAACAATTTCACACAGGA

Table S2. RNA sequences used in this study.

	Sequence:
CcCas9 crRNA	**GGG**uaucuccuuucauugagcacGUUAUAGCUCCAAUUCAGGCUCCGAUAUGCUAUAAU
CcCas9 tracrRNA	**GGG**AUUAUGGCAUAUCGGAGCCUGAAUUGUUGCUAUAAUAAGGUGCUGGGUUUAGCCCAGACCGCCAAGUUAACCCCGGCAUUUAUUGCUGGGGUAUCUUGUUUU

SpCas9 crRNA	**GGG**uaucuccuuucauugagcacGUUUUAGAGCUAUGCUGUUUUGAAUGGUCCCAAAAC
SpCas9 tracrRNA	**GGG**AACCAUUCAAAACAGCAUAGCAAGUUAAAAUAAGGCUAGUCCGUUAUCAACUUGAAAAAGUGGCACCGAGUCGGUGCUUUUUUU
CcCas9 sgRNA 1	uaucuccuuucauugagcacGUUAUAGCUCCAAUUCAGGCUCCGAUAUGCUAUAAUGAAAAUUAUGGCAUAUCGGAGCCUGAAUUGUUGCUAUAAUAAGGUGCUGGGUUUAGCCCAGACCGCCAAGUUAACCCCGGCAUUUAUUGCUGGGG
CcCas9 sgRNA 2	uaucuccuuucauugagcacGUUAUAGCUCCAAUUCAGGCUCCGAUAUGAAAAUAUCGGAGCCUGAAUUGUUGCUAUAAUAAGGUGCUGGGUUUAGCCCAGACCGCCAAGUUAACCCCGGCAUUUAUUGCUGGGG
CcCas9 sgRNA 3	uaucuccuuucauugagcacGUUAUAGCUCCAAUUCAGGCUCCGAAAGGAGCCUGAAUUGUUGCUAUAAUAAGGUGCUGGGUUUAGCCCAGACCGCCAAGUUAACCCCGGCAUUUAUUGCUGGGG
CcCas9 sgRNA 4	uaucuccuuucauugagcacGUUAUAGCUCCAAUUCAGGAAACUGAAUUGUUGCUAUAAUAAGGUGCUGGGUUUAGCCCAGACCGCCAAGUUAACCCCGGCAUUUAUUGCUGGGG
CcCas9 sgRNA 5	uaucuccuuucauugagcacGUUAUAGCUCCAAUUCAGGCUCCGAAAGGAGCCUGAAUUGUUGCUAUAAUAAGGUGCUGGGUUUAGCCCAGACCGCCAAGUUAA

DR sequences colored in red. GGG sequences – consequence of T7 RNA polymerase RNA synthesis are in bold

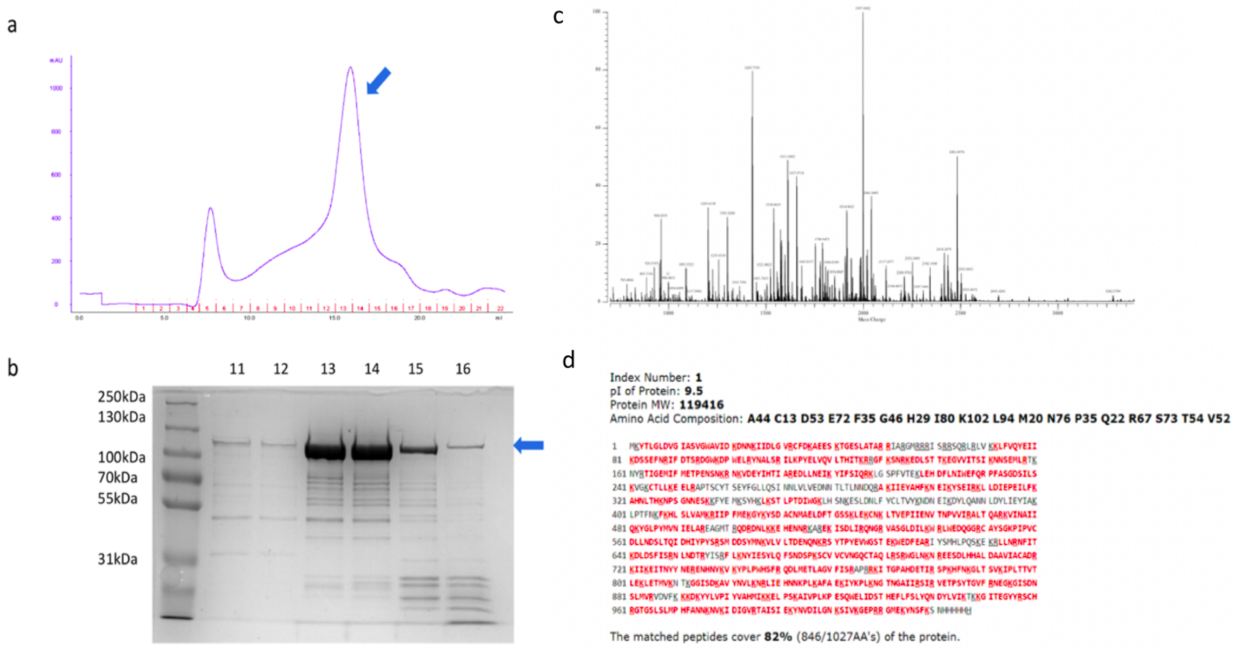

Figure S2. CcCas9 recombinant protein purification.

a. Size exclusion chromatography elution of CcCas9 protein. Monomer fraction is marked with blue arrow. The fractions numbers are written along x-axis in red.
b. SDS PAGE gel electrophoresis of size exclusion chromatography fractions 11-16. CcCas9 bands position is showed with the blue arrow.
c. Mass spectrumof tryptic hydrolyzate of gel strip corresponding to CcCas9 protein recorded by FT ICR MS (Varian) in positive MALDI mode
d. The protein sequence coverage determined by http://prospector.ucsf.edu/prospector/mshome.htm

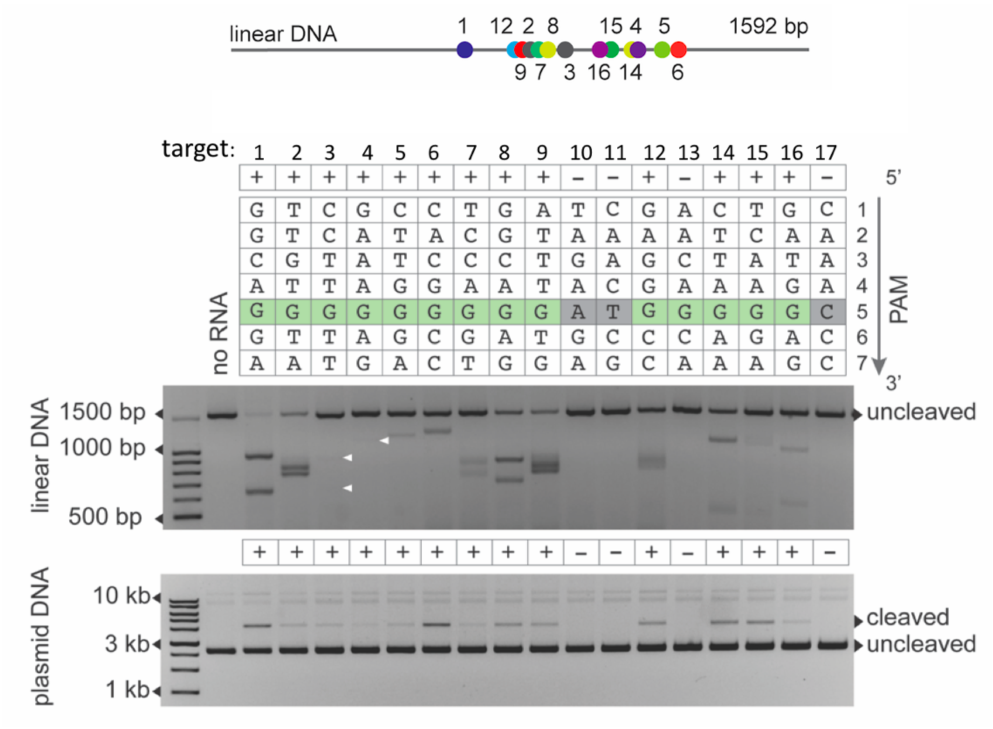

Figure S3. *In vitro* DNA cleavage of different 20-bp sites by CcCas9.
The PAM sequences corresponding to the targets are shown in the table. The +/- signs signify, correspondingly, whether cleavage was or was not observed. The conservative G in the 5th position is indicated by green color. Below two gels showing results of *in vitro* cleavage of targets with indicated PAMs on linear DNA and on plasmid DNA are presented. The white arrows indicate the positions of poorly visible bands. Above, a scheme showing the relative positions of the targeted DNA sites on the linear DNA fragment is presented.

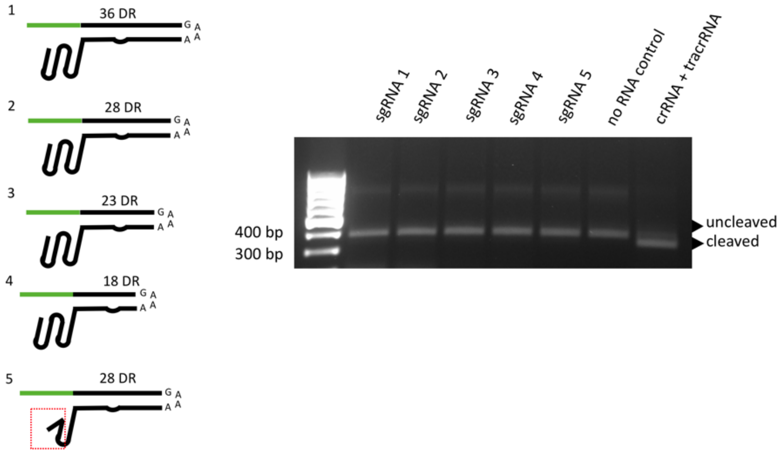

Figure S4. CcCas9 sgRNA design.
Five variants of sgRNA were used for CcCas9 DNA cleavage reactions *in vitro* (left). To estimate DNA cleavage efficiency reactions products were loaded to 1.5% agarose gel. In opposite to crRNA-tracrRNA-CcCas9 complex sgRNA-CcCas9 complexes were not able to cleave DNA targets *in vitro*. The experiment was conducted three times with three independently synthetized sgRNAs sets, which length was tested by denaturing PAGE electrophoresis.

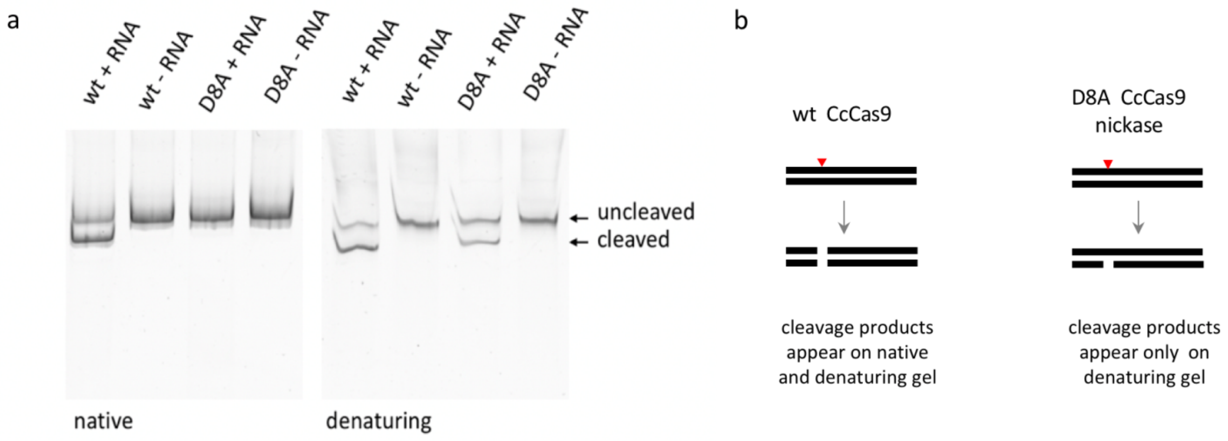

Figure S5. CcCas9 D8A mutant is a nickase.

a. wt CcCas9 protein and D8A CcCas9 DNA cleavage reactions results. Linear DNA cleavage products were loaded to native (left) or denaturing (right) PAGE gel.
b. Illustration of expectable DNA cleavage products in case of wt CcCas9 protein and a D8A CcCas9 nickase version.

Table S3. The influence of temperature on *Clostridium cellulolyticum H10* CRISPR Cas II-C locus interference.

Fractions of plasmid library members extracted from cells carrying pACYC184 (control) or pACYC184_CcCas9_locus grown at 37°C or at 22°C.

		pACYC184 carrying cells				
		37C rep1	37C rep2	37C rep3	37C mean	STD
PAM 1	ACAGGTA	0,117	0,115	0,119	0,117	0,001
PAM 2	CGGTGTA	0,178	0,176	0,175	0,177	0,001
PAM 3	TGAAGAA	0,197	0,193	0,190	0,193	0,002
PAM 4	ATTGGAA	0,263	0,264	0,265	0,264	0,001
no PAM	TTCATAT	0,246	0,252	0,250	0,249	0,002
		pACYC184_CcCas9_locus carrying cells				
		37C rep1	37C rep2	37C rep3	37C mean	STD
PAM 1	ACAGGTA	0,033	0,049	0,052	0,045	0,007
PAM 2	CGGTGTA	0,082	0,125	0,127	0,111	0,018
PAM 3	TGAAGAA	0,079	0,130	0,132	0,114	0,021
PAM 4	ATTGGAA	0,052	0,050	0,048	0,050	0,001
no PAM	TTCATAT	0,754	0,646	0,641	0,681	0,045

	pACYC184 carrying cells					
		22C rep1	22C rep2	22C rep3	22C mean	STD
PAM 1	ACAGGTA	0,118	0,117	0,120	0,118	0,001
PAM 2	CGGTGTA	0,176	0,175	0,176	0,176	0,000
PAM 3	TGAAGAA	0,189	0,191	0,193	0,191	0,001
PAM 4	ATTGGAA	0,268	0,268	0,264	0,266	0,002
no PAM	TTCATAT	0,249	0,249	0,248	0,249	0,000
	pACYC184_CcCas9_locus carrying cells					
		22C rep1	22C rep2	22C rep3	22C mean	STD
PAM 1	ACAGGTA	0,071	0,059	0,061	0,064	0,005
PAM 2	CGGTGTA	0,199	0,192	0,200	0,197	0,003
PAM 3	TGAAGAA	0,165	0,150	0,157	0,158	0,005
PAM 4	ATTGGAA	0,071	0,062	0,062	0,065	0,004
no PAM	TTCATAT	0,494	0,537	0,520	0,517	0,016

Bacterial interference experiments conducted at different temperatures: statistical analysis of the results.

ANOVA (analysis of variants) results for “no PAM” plasmid fraction:

	Df	SumSq	MeanSq	F value	Pr(>F)
temperature	1	0.0202	0.0202	17.71	0.00296 **
presence of CRISPR CcCas9 locus	1	0.3668	0.3668	322.11	9.52e-08 ***
temperature: presence of CRISPR CcCas9 locus	1	0.0198	0.0198	17.43	0.00310 **
Residuals	8	0.0091	0.0011		

The difference of “no PAM” plasmid fraction in cells carrying CRISPR CcCas9 locus comparing to control cells is significant: $F(1,8) = 322.11$ p-value = 9.52e-08 (< 0.05)

The difference between “no PAM” plasmid fraction in cells grown at different temperature is significant: $F(1,8) = 17.71$ p-value = 0.00296 (< 0.05)

ANOVA also indicates the interaction between “temperature” factor and “presence of CRISPR CcCas9 locus” factor. The effect is illustrated on graph below. The increasing of “no PAM” plasmid fraction, which illustrates the overall interference level, significantly higher in cells carrying CRISPR Cas locus grown at 37°C than at 22°C.

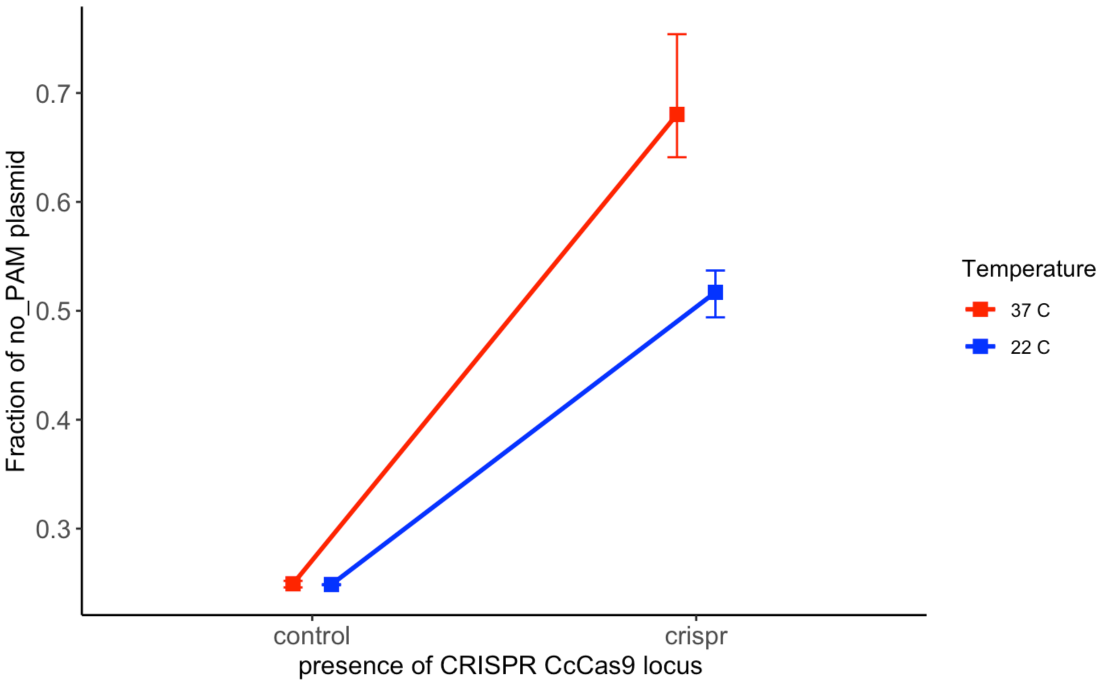

Figure S6. Fraction of “no PAM” plasmid in *E.coli* cells carrying *Clostridium cellulolyticum H10* CRISPR Cas II-C system grown at different temperatures.

A plasmid library composed of five members carrying a protospacer matching the first spacer in the CRISPR array and flanked by 5’-ACAGGTA-3’(PAM 1), 5’-CGGTGTA-3’ (PAM 2), 5’-TGAAGAA-3’ (PAM 3), 5’-ATTGGAA-3’ (PAM 4), and 5’-TTCATAT-3’ (no PAM) sequence was transformed in *E. coli* cells carrying a plasmid with the *C. cellulolyticum H10* CRISPR-Cas II-C locus or a control plasmid. Cells were plated on LB media supplemented with antibiotics, and grown for 18 h at 37 °C or 22 °C. The plasmid DNA was extracted from grown colonies and HTS was used to estimate the representation of each library member. “no PAM” plasmid fraction illustrates the overall interference level in cells. Cells carrying *Clostridium cellulolyticum H10* CRISPR Cas II-C system indicated as “crispr” and cells carrying an empty pACYC184 as “control”. The results, obtained on cells grown at 37°C and 22°C showed in red and blue color correspondingly. Mean values and standard deviations obtained from three independent experiments are shown.

Tukey multiple comparisons of means:

	diff	lwr	upr	**p adj**
22C:control-37C:control	-0.0006666667	-0.08890101	0.08756767	0.9999945
37C:crispr-37C:control	0.4310000000	0.34276566	0.51923434	**0.0000013**
22C:crispr-37C:control	0.2676666667	0.17943233	0.35590101	0.0000490
37C:crispr-22C:control	0.4316666667	0.34343233	0.51990101	0.0000013
22C:crispr-22C:control	0.2683333333	0.18009899	0.35656767	**0.0000481**
22C:crispr-37C:crispr	-0.1633333333	-0.25156767	-0.07509899	**0.0015743**

The difference of “no PAM” plasmid fraction in cells carrying the CRISPR CcCas9 locus compared to control is significant at 37 °C (p-value= **0.0000013**), as well as at 22 °C (p-value = **0.0000481**)
There is the significant difference between “no PAM” plasmid fraction in cells carrying CRISPR CcCas9 locus at 37 °C and at 22 °C (p-value = **0.0015743**).
